# Getting CLOSLER ("Closer to Osler"): Developing an Online Community Focused on Clinical Excellence

**DOI:** 10.15694/mep.2019.000052.1

**Published:** 2019-03-13

**Authors:** Charlie Stube, Margaret Chisolm, Gretchen Miller, Dean Chien, Scott Wright

**Affiliations:** 1 Oberlin College; 2 Johns Hopkins University School of Medicine; 3 Johns Hopkins University

**Keywords:** clinical excellence, Miller Coulson Academy of Clinical Excellence (MCACE), patient care, CLOSLER, medical education

## Abstract

This article was migrated. The article was marked as recommended.

Clinical excellence is the goal in healthcare delivery, at both the provider and system level. At Johns Hopkins University School of Medicine, The Miller Coulson Academy of Clinical Excellence (MCACE) was established to celebrate and promote clinical excellence in hopes of optimizing the care experienced by patients. The MCACE describes clinical excellence as achieving a level of mastery in several areas including, but not limited to, communication and interpersonal skills, professionalism and humanism, diagnostic acumen, and negotiation of the healthcare system. The Academy’s definition also puts an emphasis on teaching others to serve our patients and being a role model for clinical excellence. To have a positive influence on those beyond their immediate reach at Hopkins, the MCACE team launched *CLOSLER*, a free and open access medical education website ( closler.org). With the goal of sharing thoughtful clinical stories and perspectives to stimulate reflection about providing exceptional care to every patient, CLOSLER takes its name from William Osler, the founder of academic medicine in the United States and an early champion of patient-centered care worldwide. CLOSLER offers articles written by healthcare professionals relaying pearls and lessons from practice for all, organized into four sections: *Connecting with Patients, Clinical Reasoning, Passion in the Medical Profession, and Lifelong Learning in Clinical Excellence.* CLOSLER has ushered in a new era of education that can help to move us closer to Osler and clinical excellence; it pushes the perspectives of outstanding role models to all who are interested in developing in this realm.

## Introduction

As described in the Flexner Report ( Flexner, 1910), Johns Hopkins University School of Medicine (JHUSOM) recruited terrific clinicians as its four founding physicians (William Osler, William Henry Welch, William Stewart Halsted, and Howard Kelly), who emphasized clinical excellence and evidence-based practice from the inception. From that point on, the culture at Hopkins was one that venerated those who are most skilled and devoted to patient care. Over time at JHUSOM, similar to most other academic medical institutions, it was easier to recognize and promote our investigators. Academic centers struggle to develop methods for acknowledging those who spend a majority of their time taking care of patients. Some outstanding clinicians at Hopkins felt underappreciated and questioned whether the institution still understood and valued clinical excellence.

The Miller Coulson Academy of Clinical Excellence (MCACE) was created and launched in 2008 with the support of benefactors Sarah Miller and Frank Coulson *.* MCACE has the goal of promoting and recognizing clinical excellence for the benefit of the patients and communities served by Johns Hopkins Bayview Medical Center and Johns Hopkins Hospital. In the last decade, the MCACE’s membership has expanded to include more than 70 physicians from every clinical department. These exceptional clinician-physicians have been working together on clinical and educational programs, research, and other forms of scholarship. The members serve as inspirational role models for students and residents all across the JHUSOM campuses.

While the MCACE has served as a model for similar programs at other universities, we hoped to facilitate reflection and discussion of clinical excellence on a broad and far-reaching platform. This desire led to the development of CLOSLER; the name represents a portmanteau of the words “closer” and “Osler” - as we trust that CLOSLER can indeed help us all to become more like this legendary physician. Chief among Dr. William Osler’s beliefs was the idea that medical education and skill acquisition in medicine begins and ends with the patient. CLOSLER’s primary goal is to support a learning community that shares thoughtful clinical stories and perspectives to stimulate reflection about providing exceptional care to every patient.

## Methods

CLOSLER was meticulously designed by a creative development team, who worked with and integrated advertising agency that focuses on digital strategies. The product was constructed to look elegant and be functional on desktops, tablets, and cell phones.

CLOSLER launched in March 2018. CLOSLER is overseen by one full-time managing editor, Gretchen Miller, and many others who devote energy and attention each day to securing interesting content (particularly Drs. Margaret Chisholm & Scott Wright, MCACE manager Kim Williams, and Hopkins pre-medical student intern Dean Chien). This core team is also deliberate in their practices to expand CLOSLER’s reach.

The website posts new ideas every weekday, mostly from healthcare providers all over the country and the world, including medical students and residents / trainees in graduate medical education. Writers are encouraged to concisely share their thoughts, experiences, and perspectives on patient care and clinical practice that will help the readership to develop or upgrade their abilities. With CLOSLER, a new venue for being exposed to role models for clinical excellence was unleashed for free to anyone with Internet access.

## Examples of CLOSLER Content

Pieces on CLOSLER.org vary in length, format, organization, and cover great scope related to clinical excellence. CLOSLER divides its articles into four subcategories: *Connecting with Patients, Clinical Reasoning, Passion in the Medical Profession, and Lifelong Learning in Clinical Excellence.* Each subcategory has a separate webpage which explains the type of content that can be expected in each subcategory. Because William Osler was notoriously quotable, one of his quotes is shown at the top of each subcategory:

*Connecting with Patients*; [This section shares perspectives related to establishing deep and meaningful relationships with patients, strategies for strengthening communication and interpersonal skills, and thoughts about being consistently empathetic and responsive]:

“The good physician treats the disease; the great physician treats the patient who has the disease.”

*Clinical Reasoning;* [This section shows approaches for considering clinical scenarios or patient presentations in rigorous and systematic ways, practices for enhancing clinical judgment, and nuances to minimize the chances of making diagnostic errors and maximize the likelihood of realizing accurate diagnoses]:

“We are constantly misled by the ease with which our minds fall into the ruts of one or two experiences.”

*Passion in the Medical Profession;* [This section includes reflections on the joys and fulfillment that are encountered while practicing medicine, stories from enthusiastic role models who are inspirational, and thoughts about challenges or struggles that can be stressful] *:*

“The practice of medicine is an art, not a trade; a calling, not a business; a calling in which your heart will be exercised equally with your head.”

*Lifelong Learning in Clinical Excellence*; [This section contains descriptions of how to continually grow and develop in medicine, ideas for pulling insights from other fields into medical practice, as well as suggestions for establishing specific goals and learning plans]:

“The search for static security..is misguided. The fact is security can only be achieved through constant change, adapting old ideas that have outlived their usefulness to current facts.”

Please see Table 1 below for examples of archetypal articles that appear daily on CLOSLER.

**Table 1.** Examples of Archetypical Articles

Contributor	Title	Takeaway Message	CLOSLER Link	Section (Time stamp)
Michael Carducci, MD JHUSOM	Dr. Michael Carducci, A CLOSLER Look ( **our video series**)	Patients with cancer are vulnerable. There are a lot of emotions associated with this diagnosis, and I want to make patients feel that they are not the disease.	http://closler.org/connecting-with-patients/dr-michael-carducci-a-closler-look	Connecting with Patients (<1 min)
Samuel Durso, MD JHUSOM	How to Improve Your Physical Diagnostic Skills With a Digital Database	To sharpen diagnostic skill, take a minute to cross check the physical exam against images and lab results in the electronic record - if not congruent, then why?	http://closler.org/clinical-reasoning/how-to-improve-your-physical-diagnostic-skills-with-a-digital-database	Clinical Reasoning (3 minutes)
Lochan Shah, Neha Anand, and Priyal Gandhi JHUSOM Medical Students	Immigrants Should Not Have to Choose Between Health and a Green Card	Leverage your voice as a change agent by opposing the proposed rule so that families across the country will not have to choose between their health and their immigration status.	http://closler.org/passion-in-the-medical-profession/immigrants-should-not-have-to-choose-between-health-and-a-green-card	Passion in the Medical Profession (3 minutes)
Stephanie Cooper Greenberg Johns Hopkins Hospital Pet Therapist Volunteer	7 Things Doctors Can Learn From Therapy Dogs	Dogs see the person in front of them only as people and not as patients.	http://closler.org/lifelong-learning-in-clinical-excellence/seven-things-doctors-can-learn-therapy-dogs	Lifelong Learning in Clinical Excellence (2 minutes)
Paula Neira, JD, MSN, RN, CEN JHUSOM	Fundamentals for Caring for Transgender Patients	There are specific do’s and don’ts to most effectively support your transgender patients (and interact with your transgender colleagues).	http://closler.org/lifelong-learning-in-clinical-excellence/fundamentals-caring-transgender-patients	Lifelong Learning in Clinical Excellence (7 minutes)
Lee Randol Barker, MD JHUSOM Retiree	12 Things I Wish I’d Known 50 Years Ago	Twelve things I would tell my younger self-all the things I didn’t know then, but wish I had.	http://closler.org/lifelong-learning-in-clinical-excellence/twelve-things-wish-id-known-fifty-years-ago	Lifelong Learning in Clinical Excellence (2 minutes)
Shannon Scott-Vernaglia, MD Mass General Hospital for Children, Harvard University	Out of Office for Real	Taking a few days off for the holidays? Be a role model for your colleagues. Turn on a real out of office reply. You’ll never look back, I promise.	http://closler.org/lifelong-learning-in-clinical-excellence/out-of-office-for-real	Lifelong Learning in Clinical Excellence (4 minutes)
Patricia Dobkin, PhD McGill University School of Medicine	Becoming a Mindful Clinician	Most healthcare professionals maintain high standards, and perfectionistic tendencies. While this appears to be an advantage, these habits of mind may backfire when held too tightly - leading to emotional exhaustion. Being mindful can offset this problem.	http://closler.org/lifelong-learning-in-clinical-excellence/becoming-a-mindful-clinician	Lifelong Learning in Clinical Excellence (3 minutes)

CLOSLER aims to create unique content about clinical excellence that is found anywhere else.

First, physicians have done book reviews - both fiction and nonfiction - extracting messages that are relevant to patient care ( http://closler.org/lifelong-learning-in-clinical-excellence/book-review-of-beartown) and ( http://closler.org/lifelong-learning-in-clinical-excellence/physicians-sit-bedside-entering-patients-room).

Second, one of our regular contributors interviews authors and asks them to translate ideas from their nonmedical books into lessons that healthcare providers can use to serve their patients ( http://closler.org/lifelong-learning-in-clinical-excellence/the-dots-we-connect-an-interview-with-dan-pink).

Third, we share foundational insights into how respected clinicians approach patient care in 2 formats, (i) clinical philosophy statements ( http://closler.org/passion-in-the-medical-profession/the-power-of-laughter), and (ii) short video interviews highlighting doctors and what they keep or show in their offices - the series is called “A Closler Look” and it has its own YouTube channel ( https://www.youtube.com/channel/UCL2CyImH8fICsHrf9YGxEwg).

Fourth, clinical excellence pearls that are noted at grand rounds or other meetings are also featured on the site ( http://closler.org/clinical-reasoning/family-history-addiction-risk).

Fifth, interesting ideas about patient care that are published in biomedical journals are concisely summarized by authors with the clinical excellence take-home point distilled succinctly. For example, Dr. Gurpreet Dhaliwal published an article called Diagnostic Excellence Starts With an Incessant Watch in the *Clinical Reasoning* section of the *Annals of Internal Medicine.* This article explains how vital feedback is in refining one’s diagnostic acumen. “Feedback is the key to improvement of any craft,” says Dhaliwal. “No one is born an expert. It is earned through deliberate practice and an incessant thirst for progress.” Dhaliwal’s mentee Dr. Manesh suggests in the CLOSLER piece that every physician should have the determination and desire to master diagnosing patients ( http://closler.org/clinical-reasoning/highlights-inpatient-notes-diagnostic-excellence-starts-incessant-watch-gurpreet-dhaliwal). CLOSLER as a whole is a system that provides feedback of a certain type. Expert physicians share their experiences and lessons learned in order to teach other physicians, residents, and medical students who wish to better themselves as healthcare professionals.

Sixth, content on CLOSLER, regularly ties into the humanities. Several submissions have reminded us how art appreciation and consideration can support healthcare providers in the act of caring for patients ( http://closler.org/lifelong-learning-in-clinical-excellence/the-human-experience-of-illness and http://closler.org/lifelong-learning-in-clinical-excellence/on-looking ). Refining our observation skills ( http://closler.org/lifelong-learning-in-clinical-excellence/getting-the-most-out-of-your-eyes ) and learning from music ( http://closler.org/lifelong-learning-in-clinical-excellence/music-and-coffee) have been recently highlighted. Another piece, inspired by the showstopper from the musical Rent was called *How Do You Measure a Year?* That piece fully embodies truth that the practice of medicine being a calling to both the heart and the mind. Sherling describes in depth how people in the healthcare profession might measure a year based on quantitative information, like the amount of money generated, the number of papers published, or the number of metrics gathered. She insists, however, that a more effective and rewarding way to measure a year is by love. She reiterates that fulfillment in medicine is genuinely realized through meaningful relationships. “In 15 years of practice, I’ve found that when the patient and I have made a connection, they seem to do a whole lot better,” she says. “The more of these encounters I have, the more energy and patience I have at the end of the day. My patients are healthier and I’m happier.”

As a final example, a tease, and an invitation to join this online community, we post a “question of the week” and share almost all of the answers every Friday. Examples of some of the questions from recent weeks are: (i) “WHAT APPS HELP YOU GIVE BETTER CARE? WHAT APPS DO YOU RECOMMEND TO YOUR PATIENTS?”, (ii) “WHAT ARE YOUR SECRETS FOR ENJOYING WINTER? WHAT DO YOU RECOMMEND TO YOUR PATIENTS?”, and (iii) “WHAT’S YOUR SUPERPOWER AND HOW DOES IT HELP YOU CARE FOR PATIENTS??”. Click on the link that follows to see answers to the popular question: “What word do you wish we would delete from use in medicine and why? What would you replace it with?” ( http://closler.org/lifelong-learning-in-clinical-excellence/what-word-do-you-wish-we-would-delete-from-use-in-medicine-and-why-what-would-you-replace-it-with).

## Expansion of the Community

In our first 10 months, the number of people that have heard about CLOSLER and are engaged with this online community continues to grow. From Google analytics, we know that individuals all over the world (from over 120 countries across 6 continents) are accessing CLOSLER. Our weekly newsletter subscription list is at >8500 individuals (including medical students, residents, physicians no longer in training, nurses, other healthcare professionals, and some interested patients). While our likes on our Facebook page is growing, our primary social media platform is Twitter - where we tweet about the new content daily; we now have close to 2500 followers ( @CLOSLER).

Even more importantly, instead of needing to ask thoughtful clinicians to produce content for CLOSLER, we are now at the point where individuals are sending us great content spontaneously.

## Discussion

The desire to expand the influence of MCACE beyond our institution led to the development of CLOSLER. Our team knew that if we designed a beautiful platform (for computers and phones) and if we secured superb content that CLOSLER might be successful. CLOSLER has become an engaged online community that is focused on clinical excellence.

Just as Osler asserted that all of medicine begins and ends with our patients, CLOSLER only shares content that can help providers to become more wise, skilled, and compassionate in the service of their patients. When potential contributors pitch an idea for CLOSLER, it will not be considered unless we are convinced that the piece will make providers better in their ability to care for patients.

In the traditional medical education model, medical learners have direct exposure and experience with physicians who teach them. However, contemporary clinicians are busy and many are unable to devote as much time to educating medical trainees, leaving these learners inadequate exposure to clinically excellent role models. In this technological society, easy accessibility and automation is greatly valued by Gen Xers and millennials. Further, this next generation of medical trainees and young physicians are profoundly connected to their smartphones and keenly tied to social media. As creators of CLOSLER, we understood that digital innovations are needed to support the professional formation of these individuals. A most important part of the formation from novices to expert is learning from exemplars. In his book *Talent Is Overrated: What Really Separates World-Class Performers from Everybody Else* ( Colvin, 2008), Colvin explains how essential role-models are to becoming a world-class performer. CLOSLER expands the cohort of role models to learn from; hearing stories of success and failure from consummate clinicians from all departments facilitates learning and growth opportunities for those who may have inadequate exposure to role models in their environment. CLOSLER’s international community, with readers and contributors from all over the globe, should help to advance our understanding of cultural differences as they relate excellence in patient care.

CLOSLER was created with the sole intent of supporting those interested in thinking about and striving for clinical excellence. CLOSLER is an evolving site that is being pushed by the community of readers and contributors. We are excited to see where this journey to get closer to Osler takes us.

## Take Home Messages

Role models for clinical excellence are needed to serve as examples and inspire. CLOSLER has created a platform where such individuals can share their thoughts and experiences, thereby making clinically excellent role models accessible to all. We invite you to join and contribute to our community of clinically excellent practice.

## Notes On Contributors

Charles W. Stube is a sophomore at Oberlin College. He’s from Buffalo, New York, and is going down the pre-medical track. Stube is working to make it into medical school and hopes in the future to realize the clinical excellence that CLOSLER is preaching about.

Dean Chien is a junior in pre-medical student at Johns Hopkins University. He is from California, and is the voice of ‘A CLOSLER Look.’

Gretchen Miller is the Managing Editor of CLOSLER. She is in the process of completing her Masters in Science and Medical Writing. She and her fiancé are enjoying their new dog Rocky.

Dr. Chisolm is Professor and Vice Chair for Education in the Department of Psychiatry and Behavioral Sciences at Johns Hopkins. She is a leading academic proponent of using social media to improve clinical care. Along with Dr. Wright and others, she is co-developer of closler.org dedicated to clinical excellence.

Dr. Wright is Anne Gaines and G. Thomas Miller Professor of Medicine at Johns Hopkins. He is Chief of the Division of General Internal Medicine at Johns Hopkins Bayview Medical Center and Director of the MCACE. He serves as the Editor of CLOSLER.
