# Supplementary material for: Getting CLOSLER ("Closer to Osler"): Developing an Online Community Focused on Clinical Excellence
Source: MedEdPublish (2016). 2019 Mar 13;8:52. [Version 1] doi: 10.15694/mep.2019.000052.1 (PMC10712587; doi:10.15694/mep.2019.000052.1)
Supplement: Peer review history

# Reviewer report: Reviewer response for version 1

**DOI:** 10.21956/mep.19792.r29759

**Published:** 2019-03-24

**Authors:** James Fraser

**Affiliations:** 1 Griffith University

This review has been migrated. The reviewer awarded 4 stars out of 5 Thank you for asking me to review this paper. The authors describe the rationale and development of an online community of practice that focusses on clinical excellence in patient care. The platform curates user generated multimedia content in four broad subcategories: Connecting with Patients, Clinical Reasoning, Passion in the Medical Profession, and Lifelong Learning in Clinical Excellence. A brief view of the CLOSER website showed it to be easy to navigate and well designed. The range of articles posted in the past week “Sitting with Suffering”, “on ‘Great’ Diagnosis”, “General internal Medicine: An Opportunity to Engage Holistically”, “How do you balance efficiency in practice and providing outstanding care to all patients?” all provide varied insights that are relevant to health professionals at all stages of their careers.The relevance to clinicians across health professions is reflected by the stated membership of the subscription base. I do wonder about the classification of some subscribers as “physicians no longer in training” as we all continue to learn and train throughout our careers as reflected in the title of one of the sub-categories on the site “Lifelong Learning in Clinical Excellence”.CLOSER has developed a community by taking a unique approach to sharing skills and developments in safe and effective patient centred care. I would recommend this site to a clinicians and health professions students.

Reviewer Expertise:

NA

No decision status is available

# Reviewer report: Reviewer response for version 1

**DOI:** 10.21956/mep.19792.r29760

**Published:** 2019-03-17

**Authors:** BALAJI ARUMUGAM

**Affiliations:** 1 TAGORE MEDICAL COLLEGE AND HOSPITAL

This review has been migrated. The reviewer awarded 4 stars out of 5 Thank you so much for inviting for reviewing this wonderful new platform and learning experience article. Nothing will match the information exchange like sharing this great link which can improve peer learning. CLOSLER’s primary goal is to support a learning community that shares thoughtful clinical experiences and perspectives to stimulate reflection about providing exceptional care to every patient.The website posts new ideas every weekday, mostly from healthcare providers all over the country and the world, including medical students and residents / trainees in graduate medical education.CLOSLER aims to create unique content about clinical excellence that is found anywhere else. One of the example through the link experienced by me is on “CLINICAL REASONING | FEBRUARY 26, 2019 | 4 MIN READBy Nathan Douthit, MD, Brookwood Baptist Health, Birmingham, Alabama” – Take home message - Patients need us to excel in diagnostic reasoning. One of internal medicine’s greatest contributions to care is providing accurate and timely diagnoses. The sick need us to train and fight to make these “great diagnoses.” But we must remember their priorities. To them, diagnosis is a means to an end. The end is the relief of their pain – be it physical, mental, or spiritual. The best experience for a doctor is lifelong learning with the new technologies..CLOSLER has created a platform where such individuals can share their thoughts and experiences, thereby making clinically excellent role models accessible to all.Thanks for inviting us to join and contribute to our community of clinically excellent practice.

Reviewer Expertise:

NA

No decision status is available
